# Temperature dependent giant resistance anomaly in LaAlO_3_/SrTiO_3_ nanostructures

**DOI:** 10.1038/s41598-017-05331-y

**Published:** 2017-07-12

**Authors:** M. Z. Minhas, A. Müller, F. Heyroth, H. H. Blaschek, G. Schmidt

**Affiliations:** 1 Institut für Physik, Martin-Luther-Universität Halle-Wittenberg, Von-Danckelmann-Platz 3, 06120 Halle, Germany; 2 Interdisziplinäres Zentrum für Materialwissenschaften, Martin-Luther-Universität Halle-Wittenberg, Heinrich-Damerow-Str. 4, 06120 Halle, Germany

## Abstract

The resistance of the electron gas (2DEG) at the interface between the two band insulators LaAlO_3_ (LAO) and SrTiO_3_ (STO) typically drops monotonically with temperature and R/T curves during cooling and warm-up look identical for large area structures. Here we show that if the LAO/STO is laterally restricted by nanopatterning the resistance exhibits a temperature anomaly. Warming up nanostructures from low temperatures leads to one or two pronounced resistance peaks between 50 and 100 K not observed for larger dimensions. During cool-down current filaments emerge at the domain walls that form during a structural phase transition of the STO substrate. During warm-up the reverse phase transition can interrupt filaments before the sheet conductivity which dominates at higher temperature is reestablished. Due to the limited number of filaments in a nanostructure this process can result in a complete loss of conductance. As a consequence of these findings the transport physics extracted from experiments in small and large area LAO/STO structures may need to be reconsidered.

## Introduction

The formation of an electron gas at the interface between the two band insulators LaAlO_3_ (LAO) and SrTiO_3_ (STO) was discovered in 2004^1^. Since then its origin has been under debate and the most prominent explanations are the so called polar catastrophe^1–4^, or the presence of oxygen vacancies^5–9^. Up to now temperature dependent transport in this material system has mainly been investigated in large area structures. In these experiments the resistance typically drops monotonically upon cooling, except for a small Kondo like increase at low temperatures which is observed in some samples^10–13^. During warm-up the resistance follows the same temperature dependence as during cooling down. This behavior is in agreement with both transport models mentioned above. Also a few results for transport in nanostructures are reported. In these examples different methods were used for patterning the 2DEG. In some cases conductivity was locally induced in a non-conducting interface using a conductive atomic force microscope (AFM) tip (for example^14^). In other cases the conductivity was modulated either by the patterning of an amorphous layer on the STO substrate prior to deposition of the crystalline LAO^15^ or by low energy ion beam irradiation^16^. In all these cases, a monotonic drop in resistance with decreasing temperature was observed. Only recently an etching process was demonstrated by which the electron gas can be patterned into stable nanostructures while maintaining the interface conductivity and keeping the substrate insulating^13^. With these structures temperature dependent resistance measurements have been carried out which yield a surprising result. While the cooling curve corresponds to the one observed for large area structures the warm-up curve exhibits one or two large peaks in resistance which typically occur at the temperatures associated with structural phase transitions of the STO substrate. We have performed a series of experiments on a number of different samples in order to identify the origin of the effect.

## Results

**Figure 1:**
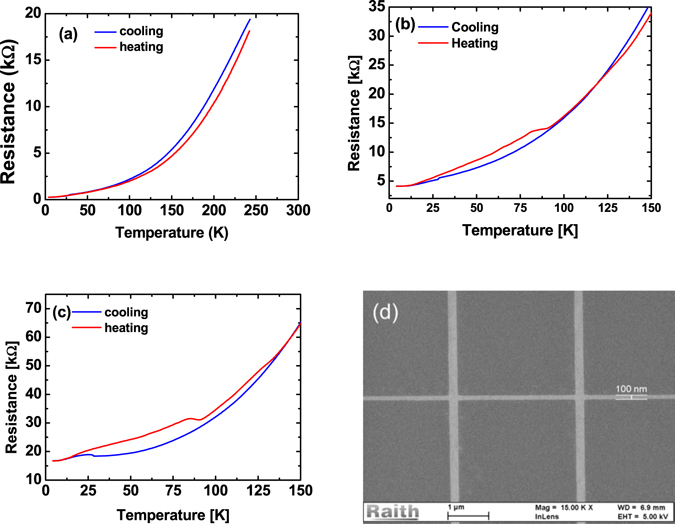
Temperature dependent resistance of the electron gas in a large Hall-bar structure (**a**), a 400 nm wide Hall-bar (**b**), and in a 200 nm wide Hall-bar (**c**). For the nano Hall-bars a non-monotonic behavior starts to appear with a resistance peak at approx. 80 K. For 400 nm width the resistance at 80 K is only about 15% higher than in the cooling curve, for 200 nm the difference is 20%. (**d**) Shows an SEM picture of a 100 nm wide LAO/STO Hall-bar.

**Figure 2:**
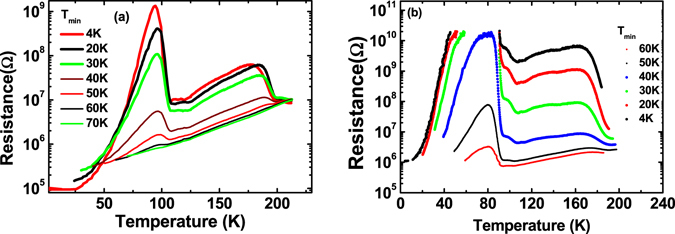
Warm-up curves for two 100 nm wide Hall-bars starting at different minimum temperatures T_min_. For lower T_min_ the peak is much higher. For the 2^nd^ sample (**b**) the resistance maximum is well beyond the measurement limit for T_min_ < 40 K.

Figure 1a shows a typical temperature dependence of the conductance of a large area Hall-bar. During cool-down the resistance decreases monotonically. During warm-up the curve is reproduced except for a small hysteresis. This artifact is due to a lag of the sample temperature with respect to the sensor. Even when the sample has been kept at low temperature for days no deviation appears. For Hall-bars with a width of less than 500 nm the cooling curve is similar to the one of a large area structure. During warm-up, however, a strong non-monotonicity is observed. At a certain temperature the resistance increases up to a maximum which can even be much higher than the resistance at room temperature (Figs. 1 and 2). When the temperature is further increased the resistance decreases again and finally the resistance curve again joins the one obtained during the cooling process. This resistance peak typically occurs at approx. 80 K. In some cases this is followed by a second smaller peak well above 100 K.

While we never observe this behavior for large area structures it is almost universal for nano-sized Hall-bars. Only the exact shape and height of the resistance peak varies from structure to structure. The effect is reproducible; however, the height of the peak depends on the history of measurements.

In order to identify the origin of this phenomenon we have done a series of investigations varying a number of parameters.

### Temperature dependence

In a first set of experiments we vary the minimum temperature (T_min_) of the cooling cycles. The sample is always kept for one hour at T_min_ before the sample is warmed up. When T_min_ is below a temperature of 60–65 K the main peak appears reproducibly almost at the same respective temperature of 80–90 K (see Fig. 2) while the increase in resistance starts already at a lower temperature. The peak height strongly depends on T_min_ and increases when T_min_ is decreased. If the minimum temperature is above approx. 60–65 K the effect vanishes completely as shown in Fig. 2. Also another important feature can be observed which will be discussed later: During warm-up the resistance decreases after the first peak, however, it does not go all the way down to the value of the cool-down curve. On the contrary there seems to be a second small peak at approx. 110 K. However, even above this temperature the resistance is still higher than during cool-down. Only at temperatures above 180 K the resistance decreases to the original value.

### Stability over time

As the time constants of the effect may give some insights into its physics we also investigate two aspects of time dependence. In a first series of experiments we vary the waiting time at the lowest temperature of 4.2 K. The minimum time is obtained by cooling down and immediately warming up when T = 4.2 K is reached, which at the cooling rates that we use corresponds to a few minutes at a temperature close to 4.2 K while the maximum waiting time is 24 hours at 4.2 K. In these experiments we do observe small variations (as is explained below the measurement history can influence the experiment) but no systematic change in peak height.

**Figure 3:**
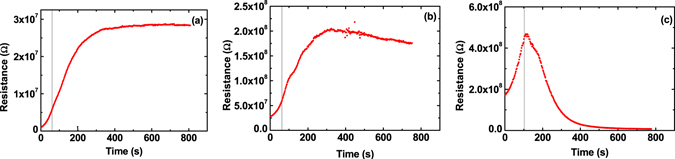
Warm-up curves for a 100 nm wide Hall-bar with waiting times at different temperatures. For (**a**) the sample is warmed from 50 K to 60 K (left hand side of grey line) and the temperature is then kept stable for approx. 12 minutes (right hand side of grey line). (**b**) Similar as (**a**) only now the sample is heated from 60 K to 70 K where the temperature is kept stable. In (**c**) the process is repeated now heating from 70 K to 80 K. While below the peak maximum (**a** and **b**) the resistance further increases while the temperature is constant beyond the peak maximum at 80 K the resistance drops over time although the temperature is constant.

In a second experiment (Fig. 3) we stop the warm-up procedure for approx. 12 minutes at 60 K and 70 K (below the peak temperature), respectively, and a third time at 80 K which for this sample is the temperature of maximum resistance. The curves obtained at 60 K and at 70 K both show the following behavior. In both cases resistance has already increased. When warm-up is stopped the resistance increase continues for several minutes and finally saturates. At 60 K the starting point of this curve is at 5 × 10^6^ Ω and the resistance increases up to 2.5 × 10^7^ Ω within 4 minutes. At 70 K the curve starts at 5 × 10^7^ Ω and increases up to 2 × 10^8^ Ω within another 4 minutes. At 80 K, however, the resistance does not increase any further but decreases massively after 10 minutes it has approximately reached the corresponding value of the cooling curve.

### Sample type

**Figure 4:**
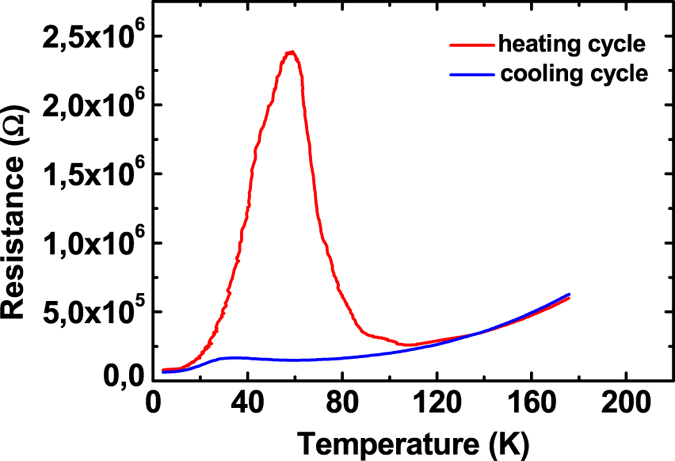
Cooling and warm-up curve for a 100 nm wide Hall-bar fabricated using the process described by Schneider *et al*.^17^. Again a large peak is observed. Even a small resistance peak is visible in the cooling curve.

As mentioned above samples have been fabricated using a dry etching process. Although we have already shown that the etch damage only influences a narrow region at the side of the nanostructures^13^ we want to make sure that the observed effect is not an artifact related to the patterning process. In order to confirm the basic nature of the observation we test additional samples fabricated using a different process introduced by Schneider *et al*.^17^. Also for these samples we see a pronounced peak as shown in Fig. 4 confirming that the lateral size restriction is the relevant factor for the increase in resistance.

## Discussion

The temperatures at which the resistance peaks occur suggest that the effect originates from the various structural phase transitions that STO undergoes during cool-down and warm-up. These phase transitions lead to the formation of structural domains in the material. A well-known transition from cubic to tetragonal occurs at approx. 110 K^18^ but another second transition at 65 K (from tetragonal to orthorhombic) is also documented in literature^18, 19^. The higher phase transition temperature is labelled T_C1_ in the following while the lower one is T_C2_. A third transition to a rombohedral phase well below 30 K is also suspected^18^. In 2013 Kalisky *et al*.^20^ showed that at low temperature the current distribution in large area LAO/STO heterostructures is no longer homogeneous but an increased current density exists along the domain boundaries in the STO substrate. In these experiments a filamentary pattern of higher current density was observed at T = 4.2 K by scanning SQUID microscopy in agreement with a typical domain pattern in the STO. The experiments, however, yielded no information on the magnitude of the conductivity of the domains themselves. At the same time Honig *et al*.^21^ identified a large anomalous piezoelectricity with a strong local variation related to the domain boundaries in the STO substrate. In 2016^20^ was succeeded by a second publication in which more detailed measurements of a similar type were presented^22^. In this work it is shown that due to the domain structure the conductivity is modulated by at least 95%. Even more recently Ma *et al*.^23^ used low temperature scanning electron microscopy and electron beam induced current for the investigation of LAO/STO structures. They not only demonstrated that highly conducting filaments exist but they also showed that these filaments may be surrounded by insulating areas. Furthermore they observed that even above the phase transition temperatures the domain wall pattern may be partially be preserved due to charging effects, an observation which is highly relevant to our findings. The presence of filaments, however, cannot be detected in transport measurements on large area structures because only the average conductivity is probed.

This filamentary current distribution alone, however, is not sufficient to explain the non-monotonic temperature dependence that we observe in nanostructures. Especially as the latter only appears during warm-up and not during cool-down additional physics needs to be taken into account.

**Figure 5:**
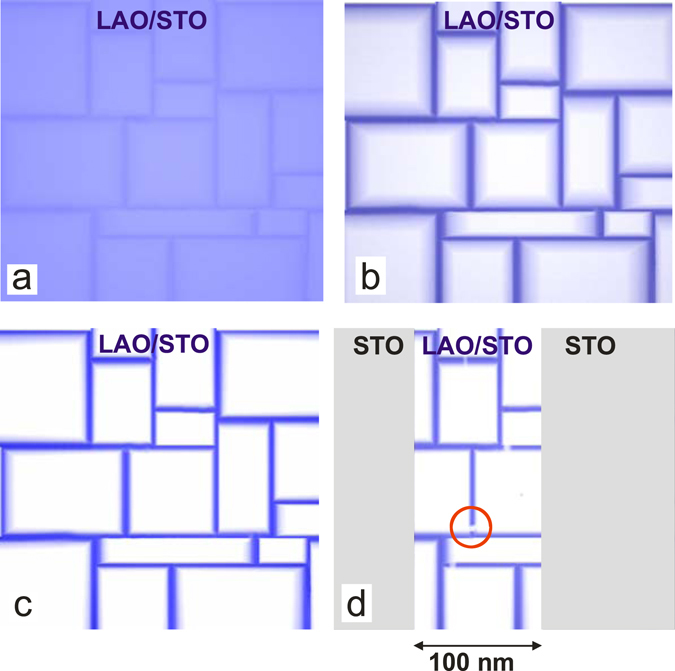
Simple sketch of a toy domain configuration which can cause the effect observed in our experiments. Dark blue regions correspond to higher conductivity, light areas have low conductivity, white is insulating. (**a**) Below T_C2_ filaments form and oxygen vacancies start to accumulate at the domain boundaries. (**b**) After some time the domain boundaries are the main conducting paths, the inside of the domains is slightly conducting. (**c**) Finally the full conductance of the sample is through the domain walls. Areas are insulating. (**d**) Situation in an etched nanostructure at warm-up. The STO (sides) is insulating while in the center (LAO/STO) filaments persist. The domain walls have disappeared; however, the vacancies have not yet been redistributed. If at a critical position a filament breaks (circle) no conductance is left.

When during cool-down temperature falls below a critical temperature (Fig. 5a) the appearing domain walls exhibit large electric fields due to the piezoelectricity and the largely increased dielectric constant at lower temperatures^24–27^. Charge and thus current flow start to accumulate at the domain boundaries as described in ref. 20. This may be accompanied by the pinning of defects at the domain walls. These defects can trap and release carriers and lead to longer time constants for charging and discharging.

Our measurements show that only after cooling to minimum temperatures below T_C2_ a resistance peak does appear during warm-up. Cooling only below T_C1_ does not induce this behavior. A possible reason is that the nature of the domain walls changes at T_C2_ and also the dielectric constant increases at lower temperatures which may additionally stabilize the charge distribution.

Below this transition the originally homogeneous conductivity in the area between the domain walls is more and more reduced while filament conductivity increases, resulting in a maze of a limited number of conducting filaments at the domain boundaries and high-resistivity (Fig. 5b) or even insulating regions (Fig. 5c) inside the domains. This corresponds to the state observed in ref. 20. Due to the way this maze is formed below the phase transition temperature there are always conducting paths. During cooling this is of no visible consequence because the total amount of charge does not necessarily change and the mobility can increase anyway leading to the typical decrease in resistance with decreasing temperature. In fact the accumulation of charge can even lead to additional screening which further reduces scattering. The effect itself is reminiscent of conducting domain walls in insulating BiFeO_3_^28^. Similarly in our samples large electric fields occur at the domain walls which can attract charges and charged defects that lead to our observations as described below.

When the sample is warmed up, the situation is different because the current is already concentrated in filaments when the phase transition temperature is approached. When the temperature is further increased not all filaments break at once. On the contrary, at some point close to T_C2_ a few domain boundaries disappear while the rest of the domain structure is still stable. As a result only a few filaments become unstable meaning that the charge is no longer pinned by local fields. The adjacent domain walls can now attract the remaining charge which was formerly pinned and thus increase the local resistance up to infinity. In large area samples this is of no consequence. With literally millions of parallel current paths, removing even thousands of them does not lead to a measurable increase in resistance. The physics of transport in filaments itself does not necessarily show up in the signatures of transport experiments, especially as the filaments are not identical and are oriented in different directions. In a nanostructure, however, the current is limited to one or at least few filaments at low temperatures (Fig. 5d).

The breaking filament then leaves a system of lower conductance if an alternative current path exists. Starting from two filaments breaking a single one just doubles the resistance. If, however, originally conduction was through a single filament the resistance may increase dramatically if not become unmeasurable. At temperatures below the phase transition this situation is stable over time as charge is still bound to the domain boundaries and the broken link cannot be mended. Only when the temperature is raised beyond T_C2_ the domain walls disappear charge becomes mobile again. Nevertheless, even above the critical temperature the original resistance value is not completely restored. For a detailed discussion it is necessary to take into account the very large increase in resistance at the peak. In several cases we observe a resistance increase of five orders of magnitude or more corresponding to an almost complete depletion. In order to decrease the resistance back by for example three orders of magnitude it is only necessary to redistribute a small fraction of the carriers. For a decrease to the original value, however, it is necessary to redistribute all carriers. Since there is no restoring force this may require a long time and temperatures elevated well above the phase transition temperature as also observed by Ma *et al*.^23^. In our case an almost complete redistribution only seems to occur above 180 K which may indicate a thermal activation. Below this temperature but above the peak temperature the warming curve merely goes parallel to the cool-down curve, however, at a higher resistance. In this range where the charge is not yet fully redistributed the phase transition at T_C1_ may also lead to a second peak in resistance (or to a small kink visible in Fig. 1c). In our experiments shown in Fig. 2 this peak is indeed observable, however, it is quite small.

Obviously the total number of filaments is bigger in bigger structures. It should, however, be noted that no direct proportionality is expected because of the statistical distribution of lateral domain size. Already from a statistical point of view this picture nicely corresponds to our observations. We have not observed any peak in resistance for large area structures. Nanostructures with a width of less than 500 nm, however, always show some peak in resistance. It should be noted that there are such observations by a number of groups^6, 29, 30^, who have observed a monotonic drop in resistance during cool-down and small non monotonicities in resistance during warm-up in a large area sample, however, the authors could give no explanation. These results will be discussed later.

We now compare this model with our additional experimental data. The finite rise time of the resistance peak (Fig. 3) is explained by the nature of the phase transition during warm-up. The phase transition happens over a certain time and temperature range. Taking into account that also the lattice contracts further during cooling or expands during warm-up, the domain structure cannot be seen as static but as something which can always undergo small modifications when the temperature changes close to T_C_. So the increase in resistance happens gradually over a certain temperature range and starts already below T_C_. Also the charges need to migrate away from the vanishing domain wall towards the remaining ones which can be a slow process. This fits the rise in resistance when warm-up is stopped at 60 K or 70 K (Fig. 3a,b) which apparently is below the phase transition in this sample. At 80 K which is above the phase transition the domain walls vanish and charges become mobile leading to a slow drop in resistance over time (Fig. 3c). The time constant of this decrease is determined by charge diffusion and by detrapping of defects because there is no other restoring force to redistribute the charge in the sample. Depending on the domains’ structure the size of the rupture and the geometry of electric fields are different and we expect a variation of peak shape and stability over time not only from sample to sample but in some cases even from measurement to measurement, especially in view of the memory effects observed by Ma *et al*.^23^.

It is important to realize that the maximum resistance is determined by the degree of depletion of the areas between filaments and not by the high conductivity of the filaments themselves. A simple gedankenexperiment shows that if the sheet conductance were reduced to 1% of the original value the resulting increase in resistance for all filaments breaking still cannot be more than two orders of magnitude. The increase observed in our case indicates quasi fully insulating domains. With this in mind the model also fits the experiments with different T_min_. Cooling to lower temperatures takes more time and leads to stronger accumulation at the domain boundaries especially because the dielectric constant of the STO increases at lower temperatures. This leaves less charge carriers for the sheet conductance at lower T. As the sheet conductance determines the peak height the peak must be higher for lower T_min_ as seen in the experiment even if the pattern of filaments is identical in all cases. The second peak which is sometimes observed (Fig. 2a) is also readily explained. When the resistance has decreased from the peak maximum above to the original value above T_C2_ this sheet conductivity is not necessarily already completely homogeneous. It only indicates that a new maze is established. The next phase transition at higher temperatures can thus again interrupt the current flow, however, only if the temperature had fallen below T_C2_ during the experiment.

It is not even self-evident that at room temperature the carriers again reach a completely homogeneous distribution. While at low temperatures the electric fields at the domain boundaries can collect the charge carriers there is no electric field except for possible Coulomb repulsion which reestablishes a uniform distribution when the domain walls are no longer present. Even when the resistance is apparently back to its original value small inhomogeneities of the charge distribution may persist. As a consequence, any further experiment can be influenced by the history of measurements and the peak height may vary even for apparently identical temperature cycles. The results are also consistent with the AFM induced conductivity mentioned in the beginning where electric fields create local conducting paths in an insulating environment which are stable up to room temperature^14^. According to the authors these paths are created by surface protonation which locally induces electric fields that can create and stabilize a kind of local domain wall.

Theoretically nanostructures may exhibit a peak in resistance even during cool-down. When the filaments are established below 80 K the third phase transition below 30 K can theoretically cause a similar scenario. Indeed for at least one structure we observe a resistance peak during cool-down below 30 K (Fig. 4) especially after several temperature cycles. Finally with this in mind also the observations of small non-monotonicities in large area structures^6, 29, 30^ can be explained. Although in these structures the effect should normally not occur it is possible that the current distribution in a large area is inhomogeneous and close to the percolation limit. In this case, the disappearance of a few filaments during warm-up can at least slightly decrease the conductance as observed in refs 6, 29 and 30. However, the statistical probability for this effect to be observed is very low.

Although the picture outlined above yields a good explanation for our observation we would like to discuss a few alternatives. As the effect appears at the phase transition temperature it is likely to be related to the domain structure and the question arises whether conducting domains and insulating domain walls might also be a suitable scenario. This can be quickly dismissed as vanishing domain walls during warm up would lead to a decrease in resistance rather than an increase. Also it is important to consider the role of defects induced by the etching process which might also change the temperature dependence of the resistance. Here a number of arguments can be setup against. Indeed a depletion region had been observed in ref. 13. This region, however, was very narrow and located at the edges of the structure. There might be an interplay of these defects with the domain boundaries, however, we would expect for a strong decrease of the effect when the width is increased from 100 nm to 200 nm, which is not observed. Also we have used two different patterning processes, one even without etching and both lead to similarly large results. It thus seems that conducting domain walls with insulating regions in between are the most likely explanation, although we are not able to determine the exact pinning mechanism.

**Figure 6:**
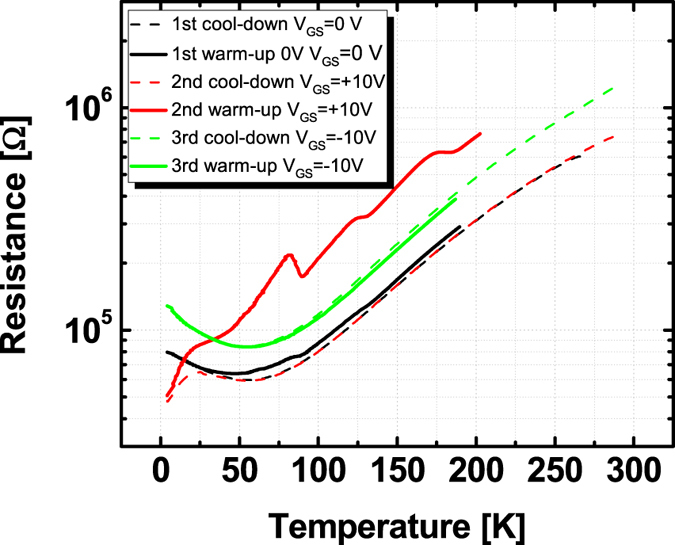
Cooling and warm-up curves taken at different back-gate-voltages. The first measurement is done at U_GS_ = 0 V. For U_GS_ = 10 V the low temperature resistance is decreased, however, the peak resistance is higher than for U_GS_ = 0 V and only at room temperature the resistance goes down to the ungated value. For negative gate voltage (U_GS_ = −10 V) the peak is completely suppressed.

Two more experiments have been carried out in order confirm our model and check for alternative explanations. On the one hand cool-down and warm-up cycles have been performed in magnetic fields of up to 2 T in order to exclude the effect of kinetic arrest^31^. On the other hand the carrier density was modulated by back gating. Both results are in line with our findings. The magnetic field has no significant influence on the curves so kinetic arrest or other influences of ferromagnetic areas can be excluded. By gating the sample we were able to change the absolute resistance of the sample, especially at low temperature. Three cool-down and warm-up cycles were performed, the first with no gate voltage, the second with positive gate voltage and the third with negative gate voltage. It should be noted that fact that we are working with nanostructures on one side of the sample and a large area back gate on the other side leads to very large electric fields at the structure despite the relatively low gate voltage. For this experiment a structure with a moderate peak height was chosen so that both positive and negative effects can be clearly observed. Figure 6 shows the results. Without gate voltage a relatively small peak is observed at the typical temperature. At positive gate voltage the low temperature resistance is lower, however, when the peak approaches the resistance increases well above the value of the measurement at zero gate voltage. The peak resistance is much higher and even above the peak temperature the resistance remains above the curve for zero gate voltage, almost up to room temperature. As in other measurements this curve now shows several peaks. For negative gate voltage the sample resistance is higher than for zero gate voltage and the peak is completely suppressed. This result nicely corresponds to our model. The appearance of the peak does not depend on the overall carrier density. It depends only on the localization of the carriers at the domain walls and the peak height depends on the carriers which remain in-between the domain walls. As Ma *et al*. have shown^23^ a positive gate voltage can increase the number of domain walls. In addition it will pull the carriers into the STO and thus into the domain walls especially at low temperatures where the dielectric constant is very high finally leaving less carriers at the interface to the LAO. As a consequence the peak height is increased and also higher temperatures are needed to restore all carriers to their original distribution. A negative gate voltage, however, not only hinders the formation of domain walls but also pushes the carriers towards the interface. When the carriers are not localized at the domain walls the domain wall dynamics no longer influence the resistance, at least not in a noticeable way.

Finally two examples of nanopatterned LAO/STO structures should be discussed which at first glance seem to contradict our findings^15, 16^. These experiments have investigated nanostructures which were not fabricated by conducting AFM and both experiments have yielded a completely monotonic dependence of resistance on temperature. There is, however, a likely explanation. Firstly Stornaiulo *et al*.^15^ have only investigated a minimum width of 500 nm which is above the threshold of 400 nm that we observe. Aurino *et al*.^16^ however, have used a minimum structure of 100 nm but with a capping layer of SrCuO_2_. Nevertheless, none of the two papers compares cool-down and warm-up curves and most likely the measurements shown were done during cool-down.

### Summary

We have shown that LAO/STO nanostructures show one or two peaks in resistance when warmed up from low temperatures. The peaks occur at the temperatures of structural phase transitions of the STO. The peak height depends on the minimum temperature of the cool-down. Above the critical temperature the maximum resistance is not stable over time but goes back to minimum after approx. 10 minutes. We can explain the behavior by the formation of current filaments at the boundaries of the domains which appear during the structural phase transition at T_C2_ in the STO and which may break during warm-up. During cool-down the formation starts from a homogeneously conducting area and is mainly undetectable. During warm-up a breaking filament can disrupt localized current paths in a nanostructure leading to the observed increase in resistance. The filaments are formed because large electric fields at the domain boundaries together with an increasing dielectric constant lead to an accumulation of charge carriers at the boundaries leaving area inside the domain basically insulating. While the effect is only observed during warm-up through the phase transition it is also of importance for the interpretation of any transport experiment in LAO/STO below T_C2_ because the assumption of a homogeneously conducting interface seems to be no longer valid.

## Methods

### Growth

All films used in our experiments are deposited by pulsed laser deposition (PLD) as described previously^13^. During growth the background oxygen pressure is 10^−3^ mbar. LAO layers are deposited from a single crystal LAO target on TiO_2_-terminated STO (001) substrates^32, 33^. The substrate temperature during deposition is 850 °C. Laser fluence and pulse frequency are kept at 2 J/cm^2^ and 2 Hz, respectively, during the deposition. Reflection high-energy electron diffraction (RHEED) is used to monitor the layer thickness with unit cell resolution during the growth. After deposition of 6 unit cells of LAO the sample is slowly cooled down to room temperature while the oxygen pressure is maintained. As a result we obtain layers with a sheet resistance of 133Ω/□  and a typical mobility of 1.124 × 10^3^ cm^2^V^−1^s^−1^ at 4.2 K.

### Nanopatterning

These layers are then patterned into nanosized Hall-bars. For this purpose two alternative processes are used in order to allow us to check for possible artifacts from processing. In the first process a resist is patterned using negative electron beam lithography. The resist is then used as an etch mask in a reactive ion etching process which removes the LAO down to the STO substrate. Details of the process have been described in ref. 13. In the second process the recipe of Schneider *et al*.^17^ is used, however, with some modifications. An epitaxial 2 u.c. of LAO is grown on STO using the same parameters as described above. Subsequently positive electron beam lithography is done and amorphous LAO is deposited and patterned by lift-off. Then a 4 u.c. layer of epitaxial LAO is deposited in an oxygen pressure of 10^−3^ mbar. Epitaxial growth, however, can only occur in the places where no amorphous LAO is present on the 2 u.c. of epitaxial LAO and the thickness of continuously epitaxial LAO exceeds 4 unit cells. As a consequence only in these areas a conducting LAO/STO interface is formed. The sample is then cooled down to room temperature in 1000 mbar of O_2_. The cool down includes a 1 h annealing step in oxygen at 600 °C. All resulting patterned structures are stable at ambient conditions. The samples are bonded and electrical transport measurements are carried out in a ^4^He bath cryostat.

### Measurements

The experiments are carried out in a ^4^He bath cryostat with a variable temperature insert. The samples are cooled down at a rate of approx. 5 K/min and warm-up is done at a rate of approx. 2.5 K/min. The resistance is measured in a four probe geometry using a nanosized Hall-bar. The active region between the voltage leads has a length of 3.5 μm and the width which is indicated in the text. The applied DC voltage is 50 mV and the current is measured using either a 100 kΩ or a 1 MΩ series resistor. Voltages are measured using custom made zero drift voltage amplifiers and an Agilent 34420 A 7.5 Digit nanovoltmeter.
